# The domestic and international implications of future climate for U.S. agriculture in GCAM

**DOI:** 10.1371/journal.pone.0237918

**Published:** 2020-08-28

**Authors:** Abigail Snyder, Katherine Calvin, Leon Clarke, James Edmonds, Page Kyle, Kanishka Narayan, Alan Di Vittorio, Stephanie Waldhoff, Marshall Wise, Pralit Patel

**Affiliations:** 1 Joint Global Change Research Institute, Pacific Northwest National Laboratory, College Park, MD, United States of America; 2 Center for Global Sustainability, University of Maryland, College Park, MD, United States of America; 3 Lawrence Berkeley National Laboratory, Berkley, CA, United States of America; Universidad Veracruzana, MEXICO

## Abstract

Agricultural crop yields are susceptible to changes in future temperature, precipitation, and other Earth system factors. Future changes to these physical Earth system attributes and their effects on agricultural crop yields are highly uncertain. United States agricultural producers will be affected by such changes whether they occur domestically or internationally through international commodity markets. Here we present a replication study of previous investigations (with different models) showing that potential direct domestic climate effects on crop yields in the U.S. have financial consequences for U.S. producers on the same order of magnitude but opposite in sign to indirect financial impacts on U.S. producers from climate effects on crop yields elsewhere in the world. We conclude that the analysis of country-specific financial climate impacts cannot ignore indirect effects arising through international markets. We find our results to be robust across a wide range of potential future crop yield impacts analyzed in the multi-sector dynamic global model GCAM.

## 1 Introduction

Research is increasingly showing that agricultural crop yields will be susceptible to future changes in temperature, precipitation, length of growing seasons, and carbon dioxide (CO2) concentrations [1–9]. While future climate is uncertain, the potential for important effects on major agricultural crop producers such as the U.S. is clear [4, 8, 10–14]. The quantity and composition of U.S. agricultural crop production, where crops are grown, trade, and economic value of U.S. crop production could be affected.

An important challenge in understanding these implications is that agricultural products are traded across the globe. The U.S. is both a major agricultural importer and exporter of agricultural crops, meaning that U.S. agriculture may be affected not only by future climate in the U.S., but also future climate outside of the U.S. via international trade. These international linkages raise questions about the relative importance of the direct and indirect effects on U.S. agriculture; that is, is the potential for changes in temperature and precipitation in the U.S. (referred to here as domestic effects) more or less important than the potential for changes outside of the U.S. (referred to here as international effects) to U.S. agricultural crop producers?

Many studies have looked at the global response of agricultural systems to changes in climate [1–3, 5, 8, 15], as well as the relative importance of where the agricultural impacts occur [10–13, 16]. In recent years, attention has begun to turn toward the implications of international trade on the U.S. agricultural sector in particular, finding that international trade effects for the U.S. agricultural sector are comparable in importance to direct, domestic impacts [10–12, 16]. Zhang et al [16] found that considering full, global climate impacts causes significant changes in the projections of U.S. production and exports of crops. Costinot et al [12] and later Gouel and Laborde [11] examined the aggregate impact of widespread local yield changes on global agricultural markets, again finding that the full picture is necessary to understand the future, as comparative advantages among regions shift with particular attention paid to domestic adjustments in land allocation. Finally, Baker et al. [10] extended these findings to a new modeling application, determining that not only does considering global markets cause significant changes in the projections of U.S. agricultural crop production relative to a U.S. only focused study, but also that freer trade may help buffer local productivity shocks. These results suggest the potential for meaningful effects on the financial revenue of the U.S, agricultural sector through changes in both domestic production and international prices.

In this paper, we systematically explore the implications of changes to domestic and international temperature and precipitation for U.S. agricultural crop production using a regionally-resolved, global scale model of energy, land, economic, and climate systems: the Global Change Assessment Model (GCAM). This paper adds to the literature by providing a complementary quantification and replication of the production effects explored in Baker et al. and other modeling efforts with a different model. The analysis in this study is conducted using a modified version of GCAM 5.2 [17] (additional documentation http://jgcri.github.io/gcam-doc/toc.html). GCAM is a global model that couples representations of the energy system, the economy, agriculture and land-use, water, and the global climate in a single computational platform.

We explore sensitivity to spatially heterogeneous future impacts by incorporating a subset of the 35 yield change scenarios from the AgMIP global gridded crop model (GGCM) intercomparison study [8] into GCAM's exogenous agricultural assumptions. The scenarios selected have been found to span a range of global impacts in previous GCAM versions [18]. Each GGCM assesses the effects on yields of RCP 8.5 changes in CO2 (including fertilization effects), temperature, and precipitation from five bias-corrected global circulation models (GCMs) for rainfed and perfectly irrigated versions of crops. Water for irrigation is constrained in the version of GCAM used for analysis. We apply crop impacts in one of two ways: (1) only in the U.S. (to isolate domestic effects) and (2) everywhere (to calculate the combined effects of direct biophysical impacts and international dynamics).

This work is consistent with earlier work using different models to consider similar scenarios regarding the importance of international impacts, adding robustness to the findings [10–12, 16] through the use of a different multisector model. We find that international impacts could be as important as domestic impacts for the financial value of U.S. agricultural crop production in across spatially and temporally varying agricultural impacts scenarios. Crucially, there are scenarios in which examining only domestic impacts would lead to a fundamentally different analysis of the future of U.S. agricultural crop production than if one considered the combined effects of domestic and international impacts. Therefore, while there is uncertainty about climate impacts on future crop yields, evidence suggests that the importance of international effects on the financial value of U.S. crop production is robust across this uncertainty.

## 2 Methods

### 2.1 GCAM background

GCAM couples human and physical Earth systems to explore the impacts of economic and environmental policies. GCAM is calibrated to historical data through 2010 and then simulates forward from 2010 to 2100 in five year timesteps by incorporating changes in quantities such as population, GDP, technology, and policy to produce outputs that include land use, emissions, and commodity prices. Specifically, GCAM can assimilate high spatial resolution information on the global distribution of crop yields and analyze its effects on the coupled system of global agriculture markets. This and previous versions of GCAM track long term trend behavior rather than interannual variability (such as price spikes). All scenarios in this study follow Shared Socioeconomic Pathway 2 (SSP2) [19], the "middle-of-the-road" socio-economic scenario. The associated GCAM scenario provides the reference against which we measure impacts in this study.

GCAM represents the energy system in 32 economic regions, and it represents global production in 384 agricultural and land-use regions. Each of the 384 land units in GCAM represents a water basin-economic region combination. Twenty-three of these lie within the U.S. With each land unit, GCAM allocates land across more than a dozen types based on cover and use. Allocation is based on a logit formulation to optimize profitability, with details provided in Wise et al [20]. Important to this study, GCAM models production of a range of agricultural commodities (crops), each with four different management types (with and without irrigation, high and low fertilizer). S1 Fig illustrates the land competition nests used by GCAM v5.2 in each GCAM land unit (updated from [20]). S1 Table in the S1 File provides the mappings between crops in the FAOSTAT database [21] and GCAM commodities. Some GCAM commodities are straightforward (Corn, Wheat, Rice), but some GCAM commodities are economic aggregates, such as MiscCrop (including high value cash crops) and OilCrop (which includes soybeans). The span of GCAM commodities allows a relatively full modeling of the agricultural sector of economies.

On the supply side of agricultural crop production in GCAM, the "no impacts" baseline yield change assumptions (derived from Food and Agriculture Organization data) are read in exogenously. These yield changes are used by GCAM to calculate the profitability of a GCAM crop-irrigation-fertilizer combination in each GCAM land unit at each time step. This profitability determines land allocated to each land type (crop-irrigation-fertilizer combination, grassland, shrubland, pasture, forest, etc.). The combination of exogenous yields and endogenous land allocation gives production of each crop-irrigation-management combination in each land unit. Because land shares allocated to rainfed versus irrigated, high versus low fertilizer versions of each crop may change, the aggregate yield for each crop output by GCAM will differ from the input yields. In other words, there is endogenous yield intensification in GCAM. AgMIP yield impacts are incorporated as multipliers on the exogenous yield assumptions used by GCAM (details in Section 2.2). The production of irrigated crops is constrained by water availability and prices (see [22–24] for more details on water supply and demand in GCAM).

The GCAM food demand system creates a slightly elastic portion of demand for each crop type, based on the exogenous population and GDP assumptions GCAM takes as inputs. Therefore, the minimum quantity defined by the food demand system must be met globally by GCAM agricultural crop production. Other demand sectors (biofuels, animal feed, non-food demand, etc.) are more elastic. For some GCAM agricultural commodities, such as Corn and OilCrop, this leads to an overall more elastic total demand function because GCAM explicitly models the energy sector and the accompanying price-sensitive demand for use of these crops as biofuels. Crops such as Wheat and Rice that are primarily used to feed humans have nearly perfectly inelastic demand. This lends an extra layer of difficulty to analysis of the dynamics in any particular agricultural impacts scenario. Since both supply and demand schedules may shift, there is never a single mechanism that may be identified for any particular price change.

GCAM includes mechanisms on both the supply and demand side that allow for adaptation behavior. Specifically, because there is a price elasticity of demand for GCAM agricultural crop commodities (albeit varying by commodity), reduction of quantity demanded is one available mechanism in the model. This includes changing demand for animal feed and bioenergy in response to changing prices. On the supply side, economic agents can endogenously adjust land allocation in response to changes in profit rates between rainfed or irrigated versions of crops (allowing changes to water consumption as a mechanism). Additionally, the option to shift to higher nitrogen fertilizer per unit of land is included, which leads to an increase in both the yield and the cost per unit of land (see [17] for details). Pesticide use is currently not explicitly modeled, and therefore changes to pesticide use are not included as an adaptation option in GCAM. Finally, GCAM includes trade of agricultural crop commodities and the ability for producers to shift land allocation among commodities as profit rates change with yield and price changes.

A significant difference between the version of GCAM used in this paper (v5.2) and the version documented in detail in [17], is that GCAM 5.2 features different agricultural crop trade behavior. Previously, GCAM modeled completely flexible trade with nearly all agricultural commodities traded freely on a global market with no explicit distinction between domestically-produced and imported products and a common global market price. The version of GCAM used for this study employs a system that is based on an Armington distinction between domestically-produced and imported products (see, e.g., [25]). In this new approach in GCAM, we specify region-specific agricultural markets at the 32-region level. Regional demand is an explicitly modeled choice between domestic production and imports from the global market via a nested logit structure, similar to our modeling of land use allocation in GCAM (Wise et al., 2014). International trade is not modeled as explicit bilateral trade but instead as a single market for each commodity that contains all regional gross imports and gross exports. Additional details may be found in the S1 File.

### 2.2 Scenario design

We examine the implications of agricultural impacts to U.S. producers through the use of four varying crop-climate model combinations in the AgMIP/ISIMIP global gridded crop model intercomparison study [8] CO2 fertilization effects, driven by RCP 8.5 earth system changes. RCP 8.5 is a climate scenario that features large changes in temperature, CO2, and precipitation and therefore relatively larger local changes in crop yields produced by crop models than under the other RCPs. It was selected with the idea that the larger yield change signal in RCP 8.5 would aid in identifying the emergent mechanisms that dictate the future economic changes resulting from yield changes. The other RCPs are deserving of future examination, but one would expect that the policies applied to reduce emissions (e.g. increased demand for biofuels) would then interact with the impacts of correspondingly smaller yield changes [22]. The GCAM reference scenario to which these crop yield impacts are applied has no climate policies in place.

**Fig 1:**
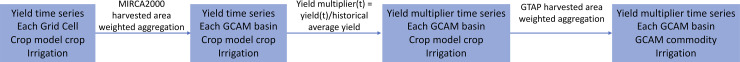
Schematic of data processing. Schematic illustrating the processing of global gridded yield time series into time series of yield multipliers for GCAM commodities in each basin under perfect irrigation and rainfed conditions.

We use AgMIP global gridded yields from each of the EPIC and LPJmL crop models driven by five GCMs under RCP 8.5. Each scenario models yields for different collections of crops. Further, these collections of crops differ from the commodities modeled in GCAM. Yield data is available for both perfectly irrigated and locally rainfed versions of each crop. These gridded yields are aggregated (separately for each crop-irrigation pair) to the GCAM land units using MIRCA2000 harvested area data for weighting [26]. At the basin scale, time series of yields are converted to multipliers by dividing by the historical baseline average yield for each crop-irrigation-basin combination. Finally, these crop-irrigation-basin specific multipliers are converted from the crop model specific crop types to multipliers for each GCAM commodity-irrigation-basin combination using GTAP harvested area weights from the GCAM data system [27] for aggregation. This method for incorporating climate driven yield changes as multipliers on GCAM's exogenous yield assumptions follows methods used in the broader literature [4, 24, 28–32]. GCAM’s biomass crop commodity receives the median of impacts to all other commodities, for each irrigation-management practice combination, in each basin. Fig 1 is a schematic summarizing this processing pipeline. S2 Table in S1 File lists the mapping between AgMIP model crops and GCAM commodities used to estimate multipliers. Corresponding water supply constraints for irrigated crops are used for each scenario [22–24]. Impacts are not applied to grassland or forest to isolate the role of impacts on crop yields.

From this suite of agricultural impacts, we construct GCAM scenarios that isolate the effect of agricultural impacts occurring outside the U.S. and impacts occurring within the U.S. For each climate-crop combinations, we run alternative variants that

1. Limit change to the U.S. alone, denoted ‘Domestic’ in results.
2. Apply the change across the whole world, including the U.S. and denoted ‘Full’ in results.

## 3 Results and discussion

**Fig 2:**
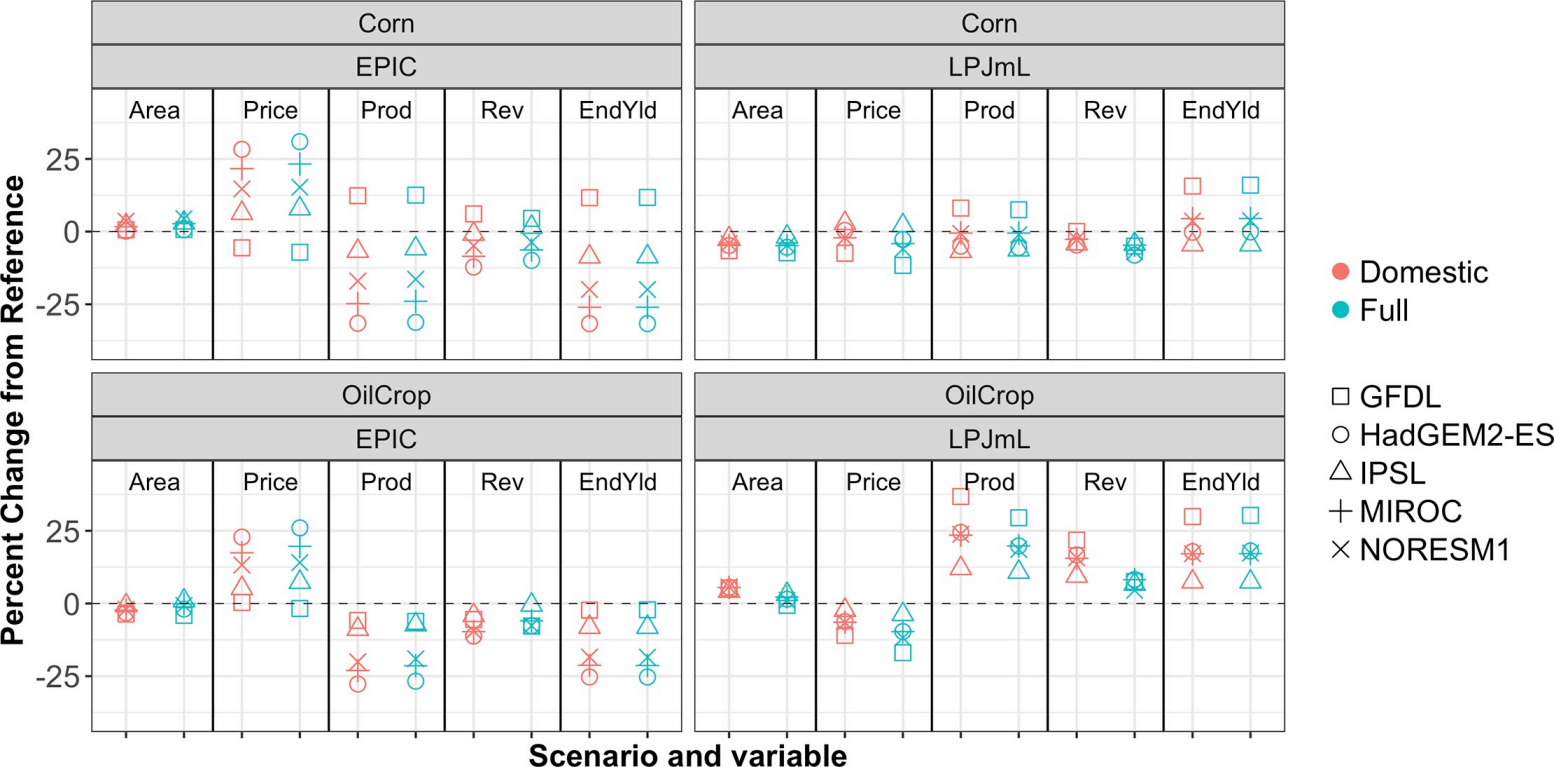
Changes in GCAM 2050 outputs for Corn and OilCrop. The 2050 percent changes from the no impacts reference GCAM scenario in the U.S. for Corn and OilCrop: Area, commodity Price, production Prod, revenue Rev, and endogenous yield.

**Fig 3:**
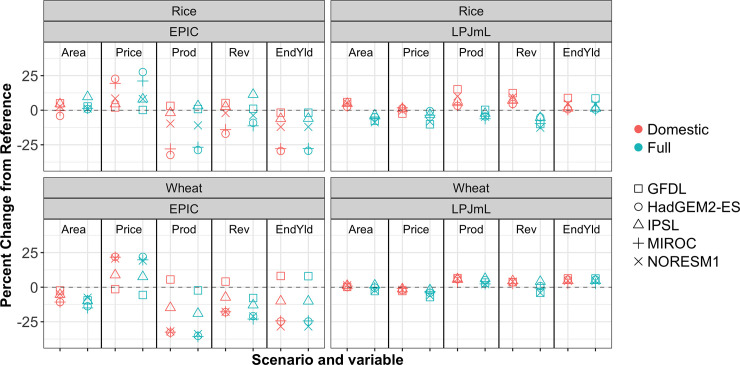
Changes in GCAM 2050 outputs for Rice and Wheat. The 2050 percent changes from the no impacts reference GCAM scenario in the U.S. for Rice and Wheat: Area, quantity demanded for animal Feed, commodity Price, production Prod, revenue Rev, and endogenous yield.

Results are presented for a variety of physical and economic output variables from GCAM. Due to both the variability and the relatively small sample size of spatially heterogeneous climate-crop impact scenarios available for this paper, summary statistics across scenarios are not presented. Rather, the results presented here focus on relationships between the Domestic and the Full scenarios that emerge in each of the different climate-crop impact combinations considered. To this end, Figs 2 and 3, and S3 Fig illustrate the percent change relative to the no impacts reference GCAM scenario for several economic and physical variables in 2050 at the aggregate U.S. level for different GCAM commodities, under for all scenarios in the work. Fig 2 focuses results on Corn and OilCrop, globally important commodities for which the U.S. is a major producer. Fig 3 presents results for Rice and Wheat, important commodities with more spatially distributed production across the globe. S3 Fig presents results for the remaining GCAM commodities. The variables plotted include *Area* allocated to each commodity (summed across management practices and GCAM land units), the U.S. commodity *Price*, the production *Prod* of each commodity (summed across management practices and GCAM land units), and the aggregate endogenous yield change *EndYld* (area-weighted averages across basin and management practices). Results are also included for changes in revenue *Rev* (total U.S. production multiplied by U.S. commodity prices) to examine an aggregate direction of change between Price and Prod, as both variables are important to U.S. producers.

Price changes are a primary economic mechanism through which international yield changes are transmitted to U.S. agricultural producers. Because agricultural commodities are traded across the globe, the prices of these commodities are affected by events that occur both in the U.S. and internationally. An event that affects U.S. yields will have consequences for U.S. production but also for prices in the rest of the world and will therefore affect production throughout the world agricultural system. The reverse also holds. Because the U.S. is a significant but not majority contributor to the global agricultural market for most crops, the magnitude of U.S. price changes in the Domestic is consistently smaller than in the Full scenario, across all climate-crop impact combinations for all GCAM commodities, illustrated in Figs 2 and 3 and S3 Fig. This is regardless of whether prices have increased or decreased relative to the baseline scenario, consistent with many of the results reported in [10,16]. Further, applying impacts in the Full case versus in the Domestic results in differing price changes from reference, even for commodities such as Corn that display very similar values in the Full and Domestic cases for physical variables such as area, production, and endogenous yield. It is possible that the relationship between the Full impacts price change from reference and the Domestic impacts price change from reference may break down as the structure of the system being modeled fundamentally changes. For example, in a more restrictive trade scenario, U.S. producers would be more restricted in their options to respond to future climate and prices would be increasingly dictated solely by the direct impacts on U.S. productivity.

For commodities such as Corn and OilCrop, for which the U.S. is a significant producer and exporter, a major shock only to U.S. production (Domestic) is closer in magnitude to a shock across the entire world (Full) than it would be for other commodities. For these commodities, the Domestic and Full changes from reference are therefore most similar. Indeed for Corn in particular, the inclusion of even spatially heterogenous, direction varying impacts across the entire world (Full) versus only in the U.S. (Domestic) makes very little difference in the physical output variables of GCAM considered: area, production, and endogenous yield. It is primarily in price (and by extension, revenue) that a difference between Domestic and Full is detectable for a given climate-crop impact combination.

For commodities such as Wheat and Rice, shown in Fig 3, for which production is more spatially distributed globally, a shock to U.S. production is a smaller change in the scope of the full global system. Therefore for commodities such as these, the inclusion of impacts globally can lead to a reversal in the direction of changes relative to the Domestic case, particularly for production and/or revenue. For Wheat, reversals in the direction of revenue change occur in three of ten spatially heterogenous agricultural impacts scenarios driven by structurally different crop models, across a range of GCM drivers; for Rice, in five of ten. This suggests that the reversal in the direction of change when impacts are applied globally is emergent from the international dynamics themselves and not an artifact of the scenarios considered. These findings are again consistent with other models in the literature, and adds an additional regionally resolved, global-scale multi-sector economic model’s results to confirm the importance of examining global systems holistically, as conclusions may fundamentally change for several commodities when only domestic impacts are considered.

Figs 2 and 3 highlight that different changes from reference in the area allocated to individual commodities occur when impacts are applied in the Domestic versus Full scenario. For a more aggregated investigation of area allocation, Table 1 summarizes the change in landcover for total cropland, as well as changes from reference in the GCAM ‘other arable land’ type, forest, and grassland cover. Recall that impacts were not applied to forest or grassland in any of the scenarios under consideration, and so these area changes are strictly emergent from the changes in cropland as areas move into or out of agricultural crop production. The changes in forest and grassland are generally small, as GCAM features an explicit other arable land type in the crop competition logit nest (see Fig 1 and [20] for details). In the system modeled by GCAM, this land type is often the first and most impacted by cropland area changes. An exception is the HadGEM2-EPIC scenario: cropland areas decrease to such an extent that both other arable land and forest expand. The changes in total cropland area in the EPIC scenarios reported in Table 1 are generally smaller in magnitude than those reported in [10].

**Table 1:** 2050 percent changes from the no impacts reference GCAM scenario in the U.S. for different land cover types under different impacts scenarios.

		Total Cropland		Other Arable Land		Forest		Grass, Shrub, Pasture	
Model	GCM	Domestic	Full	Domestic	Full	Domestic	Full	Domestic	Full
**EPIC**	**GFDL**	-0.95	-2.92	1.11	4.42	0.42	0.73	0.01	0.25
**EPIC**	**HadGEM2-ES**	-5.57	-4.57	5.00	1.57	2.31	2.62	0.22	-0.04
**EPIC**	**IPSL**	-0.89	-0.31	-1.44	-3.64	0.73	0.95	-0.01	-0.17
**EPIC**	**MIROC**	-4.31	-3.76	4.37	3.20	1.66	1.65	0.20	0.11
**EPIC**	**NORESM1**	-1.84	-1.81	4.05	4.11	0.34	0.30	0.14	0.14
**LPJmL**	**GFDL**	1.12	-3.10	-1.93	6.53	-0.15	0.16	-0.15	0.47
**LPJmL**	**HadGEM2-ES**	0.97	-1.16	-2.54	1.82	0.04	0.19	-0.16	0.15
**LPJmL**	**IPSL**	1.43	0.75	-2.53	-0.50	-0.20	-0.32	-0.18	-0.03
**LPJmL**	**MIROC**	1.06	-0.67	-2.90	1.14	0.00	-0.01	-0.15	0.14
**LPJmL**	**NORESM1**	1.49	-2.04	-2.37	5.04	-0.29	-0.11	-0.17	0.37

While GCAM’s simulations run through 2100, results are presented in 2050 for ready comparison with other results in the literature. S4 and S5 Figs present the 2100 data for the same variables as Figs 2 and 3 and S3 Fig; the relationships between the Domestic and Full scenarios that occur for each climate-crop combination observed above for 2050 data persist in 2100. The persistence of the relationships between Domestic and Full scenarios across time and across spatially heterogenous, varied climate-crop combinations again highlights the importance of accounting for international dynamics in examining agricultural quantities.

## 4 Conclusions

Even given the uncertainty in future agricultural changes, the importance of international effects on U.S. agricultural crop production and prices is robust across the widely varying scenarios studied here. Specifically, in many of the scenarios considered, the inclusion of impacts globally reverses the projected direction of change from reference compared to applying impacts only domestically for several key agricultural commodities. These findings are again consistent with other models in the literature, and adds an additional regionally resolved, global-scale multi-sector economic model’s results to confirm the importance of examining global systems, particularly agricultural systems, holistically, as conclusions may fundamentally change when only domestic impacts are considered.

At the same time, more research on the issues explored in this paper is needed to develop a deeper understanding of how future climate may affect the agricultural system across the globe. There are opportunities for future work across both the human and the Earth system sides of agriculture. For example, the type of analysis in this paper could be applied to any country or region of interest that is coupled to international agricultural commodity markets. In addition, our examination has focused on the producers of agricultural products. We have not attempted to analyze the effects of external and domestic changes in climate on consumers, whose interests are different and deserving of separate analysis. Next, a wide variety of different trade patterns and regimes could emerge over the long time period of this study, which could lead to different results. For example, in the absence of flexible trade, or in a more restrictive trade scenario, the balance of domestic effects would become increasingly important. Another area of future research is to quantify the effect of different socioeconomic pathways on the results, through a systematic repetition and comparison of this experiment across the SSPs. Additionally, we have focused on the effects of climate change on crops, but applying impacts to livestock agriculture in GCAM and examining the effects of those impacts would be interesting. There is evidence [33] that the integration of livestock impacts may lead to more complex results, as different effects offset or feedback on each other. Similarly, the same framework for analysis could be applied to scenarios with different energy or agricultural policies or different RCPs. Because GCAM includes both energy and agriculture modules, an increased demand for biofuels in the energy sector will bolster prices for several crops in conjunction with climate derived changes in prices.

From a more biophysical perspective, this study has explored the impacts of changing trends in agricultural yields. Consideration of extreme events such as droughts, might add a more a more dynamic and varied character to the results from year-to-year. While GCAM includes a reference assumption of increasing yet saturating yields out to 2100 to account for technological development, scenarios explicitly detailing the introduction of more drought-resistant cultivars could provide an additional dimension to this analysis. As the modeling capabilities to analyze such scenarios come online, including dynamic, two-way feedbacks between the Earth system and agriculture, will also push future analyses to provide a fuller understanding of the interactions of the different sectors of the human-Earth system.

## Supporting information

S1 File (DOCX)

S2 File (DOCX)

S3 File (DOCX)

S1 Fig (TIFF)

S2 Fig (TIFF)

S3 Fig (TIFF)

S4 Fig (TIFF)

S5 Fig (TIFF)

S1 Data (ZIP)

S2 Data (ZIP)
