# Supplementary material for: The domestic and international implications of future climate for U.S. agriculture in GCAM
Source: PLoS One. 2020 Aug 28;15(8):e0237918. doi: 10.1371/journal.pone.0237918 (PMC7455037; doi:10.1371/journal.pone.0237918)
Supplement: S1 File — (DOCX) [file pone.0237918.s001.docx]

## S1: GCAM land allocation and crop mappings

**S1 Fig. The GCAM land nest structure.** The land nest structure used by GCAM in each land-use region for land allocation. Corn is highlighted as an example to illustrate the four management types for each agricultural commodity within the ‘Corn, wheat, bioenergy, etc.’ nest. The GCAM commodities within the ‘Corn, wheat, bioenergy, etc.’ nest are listed in S1 Table with the crops from the FAOSTAT database that are assigned to each GCAM commodity.

**S1 Table: The mappings between crops in the FAOSTAT database and the GCAM agricultural commodities.**

| **GCAM Category** | **Crops Included** |
| --- | --- |
| Corn | Maize; Maize, green; Popcorn |
| FiberCrop | Agave Fibres Nes; Cotton; Fibre Crops Nes; Flax fibre and tow; Hemp Tow Waste; Jute; Manila Fibre (Abaca); Other Bastfibres; Ramie; Sisal |
| FodderGrass | Forage Products; Grasses Nes for forage; Rye grass for forage and silage; Sorghum for forage and silage |
| FodderHerb | Alfalfa for forage and silage; Beets for Fodder; Cabbage for Fodder; Carrots for Fodder; Clover for forage and silage; Green Oilseeds for Silage; Leguminous for Silage; Maize for forage and silage; Swedes for Fodder; Turnips for Fodder; Vegetables Roots Fodder; Vetches |
| MiscCrop | Almonds, with shell; Anise, badian, fennel, corian.; Apples; Apricots; Arecanuts; Artichokes; Asparagus; Avocados; Bambara beans; Bananas; Beans, dry; Beans, green; Berries Nes; Blueberries; Brazil nuts, with shell; Broad beans, horse beans, dry; Cabbages and other brassicas; Carobs; Carrots and turnips; Cashew nuts, with shell; Cashewapple; Cauliflowers and broccoli; Cherries; Chestnuts; Chick peas; Chicory roots; Chillies and peppers, dry; Chillies and peppers, green; Cinnamon (canella); Citrus fruit, nes; Cloves; Cocoa beans; Coffee, green; Cow peas, dry; Cranberries; Cucumbers and gherkins; Currants; Dates; Eggplants (aubergines); Figs; Fruit Fresh Nes; Fruit, tropical fresh nes; Garlic; Ginger; Gooseberries; Grapefruit (inc. pomelos); Grapes; Hazelnuts, with shell; Hops; Kiwi fruit; Kolanuts; Leeks, other alliaceous veg; Leguminous vegetables, nes; Lemons and limes; Lentils; Lettuce and chicory; Lupins; Mangoes, mangosteens, guavas; Mate; Mushrooms and truffles; Nutmeg, mace and cardamoms; Nuts, nes; Okra; Onions (inc. shallots), green; Onions, dry; Oranges; Other melons (inc.cantaloupes); Papayas; Peaches and nectarines; Pears; Peas, dry; Peas, green; Pepper (Piper spp.); Peppermint; Persimmons; Pigeon peas; Pineapples; Pistachios; Plantains; Plums and sloes; Pulses, nes; Pumpkins, squash and gourds; Pyrethrum,Dried; Quinces; Raspberries; Sour cherries; Spices, nes; Spinach; Stone fruit, nes; Strawberries; String beans; Tangerines, mandarins, clem.; Tea; Tea Nes; Tobacco, unmanufactured; Tomatoes; Vanilla; Vegetables fresh nes; Walnuts, with shell; Watermelons |
| OilCrop | Castor oil seed; Groundnuts, with shell; Hempseed; Jojoba Seeds; Karite Nuts (Sheanuts); Linseed; Melonseed; Mustard seed; Oilseeds, Nes; Olives; Poppy seed; Rapeseed; Safflower seed; Sesame seed; Soybeans; Sunflower seed; Tung Nuts |
| OtherGrain | Barley; Buckwheat; Canary seed; Cereals, nes; Fonio; Millet; Mixed grain; Oats; Quinoa; Rye; Sorghum; Triticale |
| PalmFruit | Coconuts; Oil palm fruit |
| Rice | Rice, paddy |
| Root_Tuber | Cassava; Potatoes; Roots and Tubers, nes; Sweet potatoes; Taro (cocoyam); Yams; Yautia (cocoyam) |
| SugarCrop | Sugar beet; Sugar cane; Sugar crops, nes |
| Wheat | Wheat |

**S2 Table: Mapping between GCAM commodity and AgMIP crop. Note: multiple entries in the same cell indicate that several AgMIP crops are used to estimate the response of a given GCAM commodity, with weightings across crops given by harvested areas.**

| **GCAM Commodity** | **EPIC** | **LPJmL** |
| --- | --- | --- |
| biomass | median of all crops | median of all crops |
| Corn | Maize | Maize |
| FiberCrop | Cotton and C3 average | C3 average |
| FodderGrass |  |  |
| FodderHerb | Maize, Sorghum, and C3 average | Maize, Sorghum, and C3 average |
| MiscCrop | Groundnut, Drybean, and C3 average | Groundnut, Field pea, and C3 average |
| OilCrop | Soybean, Rapeseed, Sunflower, Groundnut and C3 average | Soybean, Rapeseed, Sunflower, Groundnut and C3 average |
| OtherGrain | Barley, Millet, Sorghum, Wheat, and C3 average | Barley, Millet, Sorghum, Wheat, and C3 average |
| PalmFruit | C3 average | C3 average |
| Rice | Rice | Rice |
| Root_Tuber | Casava and C3 average | Casava and C3 average |
| SugarCrop | Sugarcane and Sugarbeet | Sugarcane and C3 average |
| Wheat | Wheat | Wheat |
