# Supplementary material for: The domestic and international implications of future climate for U.S. agriculture in GCAM
Source: PLoS One. 2020 Aug 28;15(8):e0237918. doi: 10.1371/journal.pone.0237918 (PMC7455037; doi:10.1371/journal.pone.0237918)
Supplement: S2 File — (DOCX) [file pone.0237918.s002.docx]

## S2: Agricultural Trade Structure

The version of GCAM used in this analysis (version 5.2) differs from GCAM versions 5.1 and previous versions in its handling of international trade in agriculture. GCAM as used here uses a gross trade model with differentiated regional prices with an explicitly modeled choice between consumption from domestic production and from imports from the global market. (Previous versions of GCAM agricultural trade were based on a Heckscher Ohlin approach where there is one single global market per commodity with all regions sharing a common global price, and only net trade is modeled.) S1 Fig shows an example of the trade structure used in this study. From the figure, regional demands (or consumption) are differentiated from regional production and imports from the traded sector, while regional production goes to a mix of regional domestic supply and exports to the traded sector.

**S2 Fig. Example GCAM trade structure.** Example GCAM trade structure for the flows of the "Corn" commodity in GCAM as an example, with only 3 regions shown for simplicity.

For each commodity, historical quantities are calibrated to data based on each region's gross exports to countries that are not within the same GCAM region. The data relies on FAOSTAT's bilateral trade matrix. In S2 Fig, the items called "Corn Domestic Supply" explicitly compete imports of globally traded corn with local production. This decision is calibrated in the historical years, and in the future years is responsive to changes in the prices of these two options, each of which are explicitly represented. In this representation, there are two logit-sharing competitions that determine each region's trade balances in future years: (1) the competition between imports and domestic production, and (2) the competition between all GCAM regions in supplying to the global traded markets. Note that due to structural changes and differentiated prices of the modeled commodities, there is no set of logit exponents that could replicate the prior model's behavior.

The approach in distinguishing domestically produced and imported commodities is based on an Armington approach that is prominent in several models of international trade (see, e.g.[25]). Where the GCAM approach used here differs from other Armington implementations is that we do not follow a bilateral approach. Instead we assume that all international trade is through one common global market for each commodity.

### S2.1 Regional Prices

Data on regional prices come from FAOSTAT [21], which reports producer prices by country of a large number of crop and animal commodities from 1991 to 2012 in nominal US dollars per tonne. Prices are converted from nominal to real US dollars using FAO's GDP deflators, using a base year of 2010. FAO crops are weighted by production volumes for aggregation to GCAM's commodity classes. After the weighted average prices for each year have been determined, the 5 years between 2008 and 2012 are averaged, unweighted, to get our calibration prices for the 2010 model base year. The purpose of using the unweighted average for the years is to avoid potential bias from more productive years having relatively high weights in this calculation, while also having lower prices.

Because the dataset is heterogeneous in coverage, with many missing country/crop/year combinations, there are some gap-filling steps. Simple omissions of crops within regions are filled by using the global average price for each given crop multiplied by the region's average producer price index. Each region's average producer price index is computed as the production-weighted average price index of all commodity classes, where each commodity's price index is computed as the region's price divided by the global average. The purpose of indexing prices prior to performing any cross-commodity calculations is to avoid the distortion from the commodity composition; i.e., that regions that produce more expensive crops would have a higher price index when computed over all crops. In this way, any region's producer prices for all commodities can be filled out, regardless of how many were missing in the source data. That is, even regions that don't produce a given commodity (e.g., Canada PalmFruit) are nevertheless assigned (imputed) producer prices.

An additional set of modifications is performed for cotton, which has up to three reported primary commodities in FAOSTAT: cotton seed, cotton lint, and seed cotton (the latter refers to the whole crop, prior to ginning). Our approach in general for this crop is to estimate the total revenue divided by the total production volume (seed + lint), assuming FAO's default lint:seed ratios. However, the reporting is heterogeneous, and because cotton lint is about 7 times more valuable than cotton seed (in the USA), this heterogeneous reporting can be problematic. For example, countries that only report cotton seed prices would have very low estimated cotton (and therefore FiberCrop) prices, if no effort were made to estimate the cotton lint price. The approach taken is as follows: for countries that report cotton lint but not cottonseed prices, the cottonseed prices are estimated as the lint price times the seed:lint price ratio for the USA (about 1/7). However, for countries that only report cottonseed prices, these prices are not used to estimate lint prices; instead, the cotton seed price is not considered, and the whole commodity's (FiberCrop) average prices are estimated from the global average multiplied by the regional index described above. This is because cottonseed is a by-product of cotton lint production (selling seed accounts for <10% of the revenue of the whole operation in the USA, according to USDA), and its prices are considered to be an unreliable indicator of cotton lint prices.

### S2.2 Gross Trade

Data on gross trade by region is from FAOSTAT's bilateral trade matrix, which reports trade flows between "reporter countries" and "partner countries" for a large number of commodities from 1986 to 2012. In our approach, we explicitly represent gross trade only in the final calibration year; all prior model time periods simply report the net trade, which has always been available in all versions of GCAM. This approach is taken because (a) the full historical time series from 1971 is not available; and (b) such data from prior historical years are not necessary for constructing calibration data for the model and would not affect the future projections. Note that one can still run in hindcast mode with a base year of 1990 by setting the ag trade calibration years to that interval.

In the bilateral trade data, for any typical year and crop, the sum of global imports are up to 30% different from the sum of global exports. Our methods do complete the data to some extent, described below. Nevertheless, our methods assign precedence to the net trade volumes from prior versions (based on the commodity balances, not the bilateral trade matrix), and use the bilateral trade matrix to (a) separate our existing estimates of net trade into gross imports and exports, while (b) filtering out any trade that occurs between countries that are in the same GCAM region. For example, trade between EU-15 member states is filtered out of our estimates of gross imports and exports. This sort of filtering is only possible from bilateral trade data.

Regarding data gap filling, there are a number of countries in the bilateral trade matrix that are listed as "partner" countries but not "reporter" countries. For example, Vietnam does not report any trade, but appears as a "partner" to many other nations that do report trade. In such cases of asymmetry, where a country does not report its trade, we use their trade as a "partner" country to build their trade balances. At this point, the GCAM regions are joined in to the database, and any trade where the reporter country is the same as the partner country is dropped.

Finally, the gross trade volumes are used to disaggregate net trade (from the commodity balances) into gross import and export components. This is done with the following rules:

1. If the region is a net exporter: Scaled Gross Exports = Unscaled Gross Exports + (Scaled Net Exports - Unscaled Net Exports)
2. If the region is a net importer: Scaled Gross Imports = Unscaled Gross Imports + (Scaled Net Imports - Unscaled Net Imports)

Note that these imbalances could be reconciled through multiplicative scaling, and early versions of this development did try that, but the approach was found to greatly distort trade quantities in selected cases, particularly where the two datasets (commodity balances and bilateral trade matrix) differ in estimates of net exports. The method adopted only changes one of the two gross trade flows in each region (i.e., either imports or exports), and applies the minimum possible modification to the gross trade data in order to conserve the net trade flows computed from the commodity balances.

One final data adjustment is necessary here: there are some GCAM regions that act as pass-through terminals, which import and subsequently export the same commodity. For example, the USA imports palm oil ("PalmFruit"), and subsequently exports some portion thereof to other nations. This gross quantity exported is greater than what is produced domestically. In our modeling structures (described below), one of the quantities calibrated in building each region's commodity balances is own consumption of domestic production. Such a scenario would yield a negative estimate of this quantity, which is computed as production - exports (also equal to total domestic supply minus imports). Because GCAM can not have negative calibration quantities, the approach taken is as follows: if a region exports more than it produces, all domestic production is assumed to be consumed domestically, gross scaled imports are set equal to scaled net trade, and gross scaled exports are set to 0. The following section documents the structural changes to the model that accommodate these trade flows.
