# Supplementary material for: The domestic and international implications of future climate for U.S. agriculture in GCAM
Source: PLoS One. 2020 Aug 28;15(8):e0237918. doi: 10.1371/journal.pone.0237918 (PMC7455037; doi:10.1371/journal.pone.0237918)
Supplement: S3 File — (DOCX) [file pone.0237918.s003.docx]

## S3 GCAM outputs for additional commodities.

**S3 Fig. GCAM 2050 output variables for other commodities**. The same variables presented as Figs 2 and 3, but for other GCAM commodities in 2050.

**S4 Fig. GCAM 2100 output variables for Corn, OilCrop, Rice, and Wheat.** The same variables for the same commodities presented as in Figs 2 and 3, but for 2100 data rather than 2050.

**S5 Fig. GCAM 2100 output variables for other commodities**. The same as S3 Fig, but for 2100 rather than 2050.
