# Supplementary figures and images for: The domestic and international implications of future climate for U.S. agriculture in GCAM
Source: PLoS One. 2020 Aug 28;15(8):e0237918. doi: 10.1371/journal.pone.0237918 (PMC7455037; doi:10.1371/journal.pone.0237918)

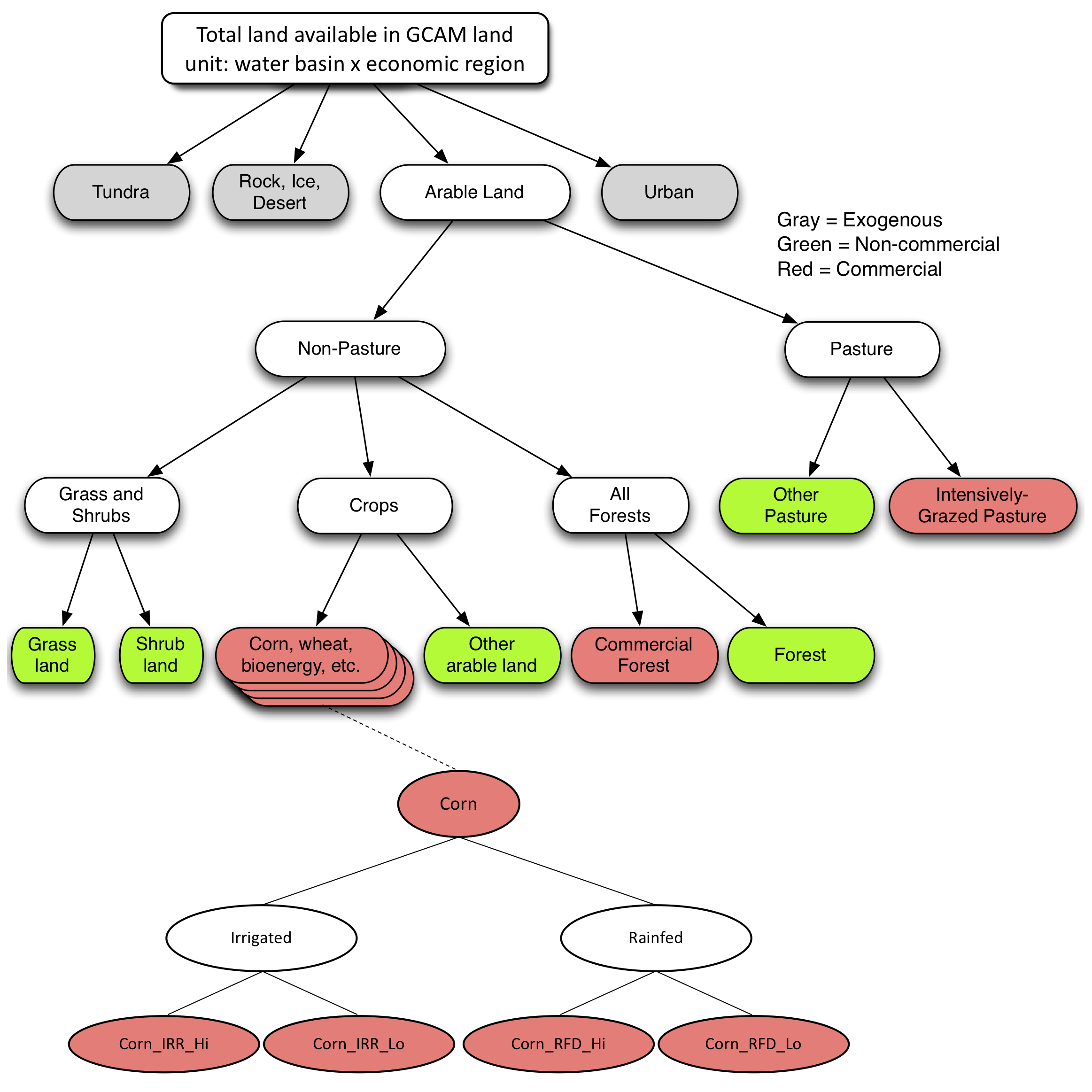

Supplement: S1 Fig — (TIFF) [file pone.0237918.s004.tiff]

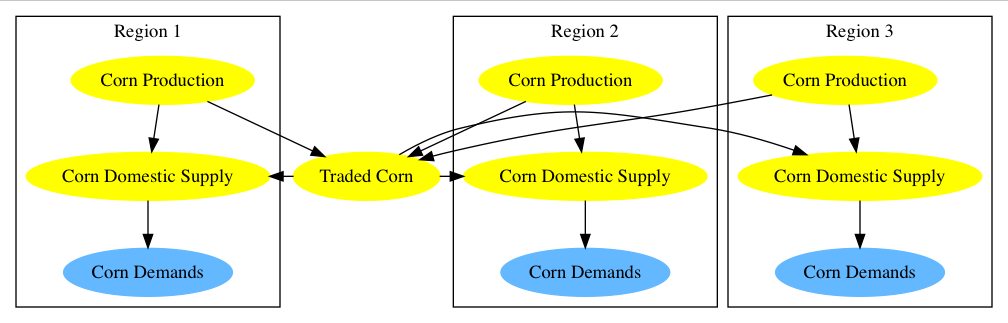

Supplement: S2 Fig — (TIFF) [file pone.0237918.s005.tiff]

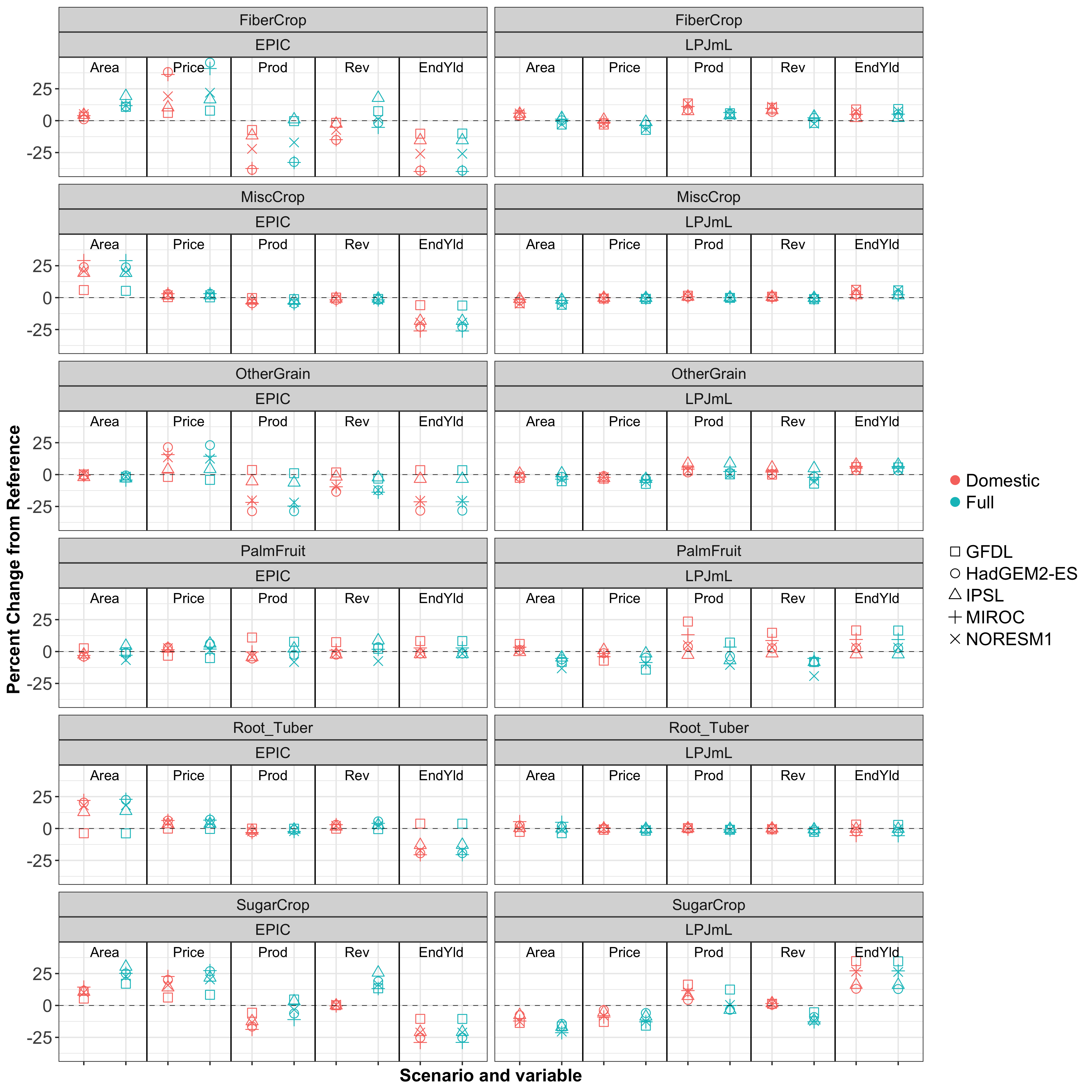

Supplement: S3 Fig — (TIFF) [file pone.0237918.s006.tiff]

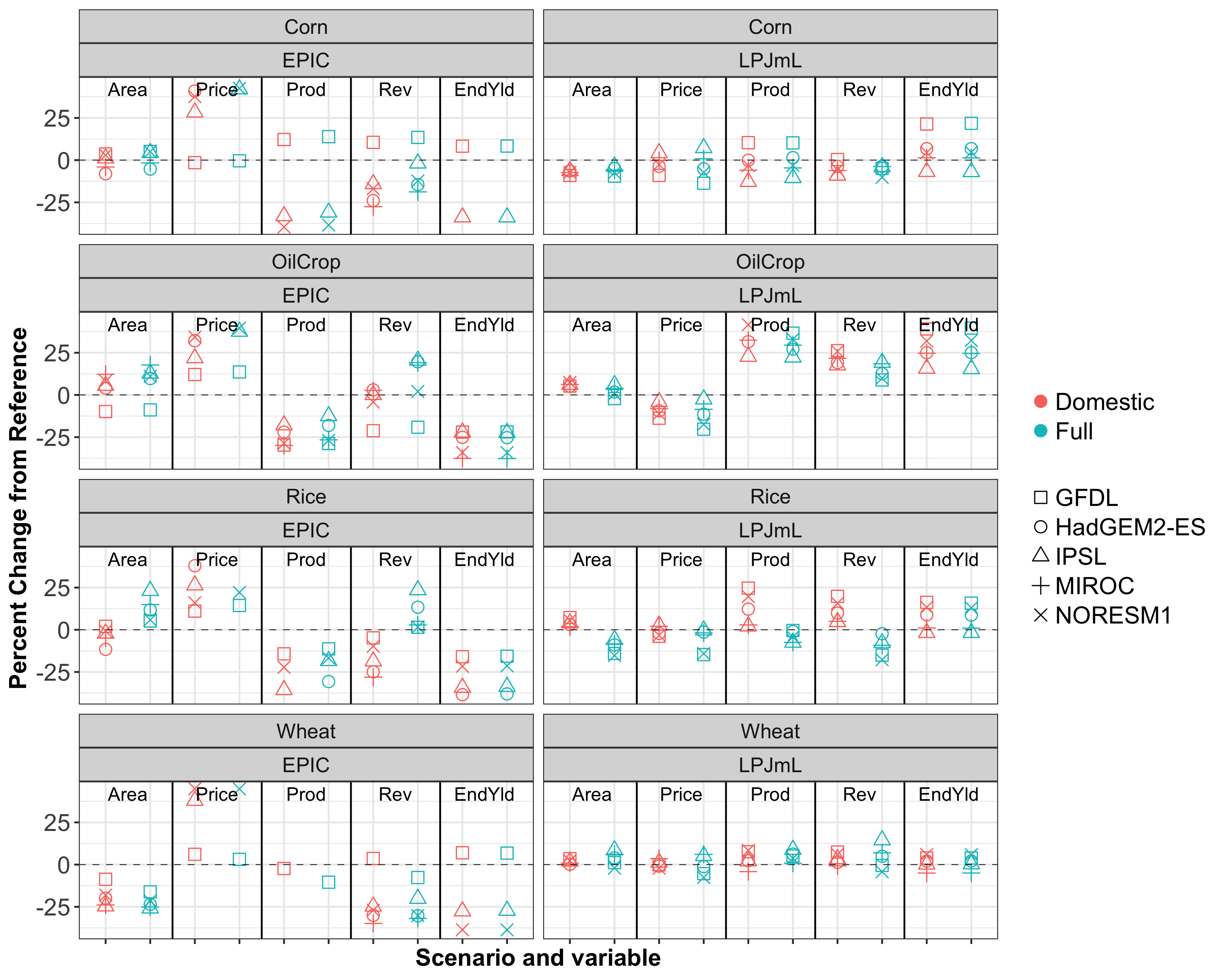

Supplement: S4 Fig — (TIFF) [file pone.0237918.s007.tiff]

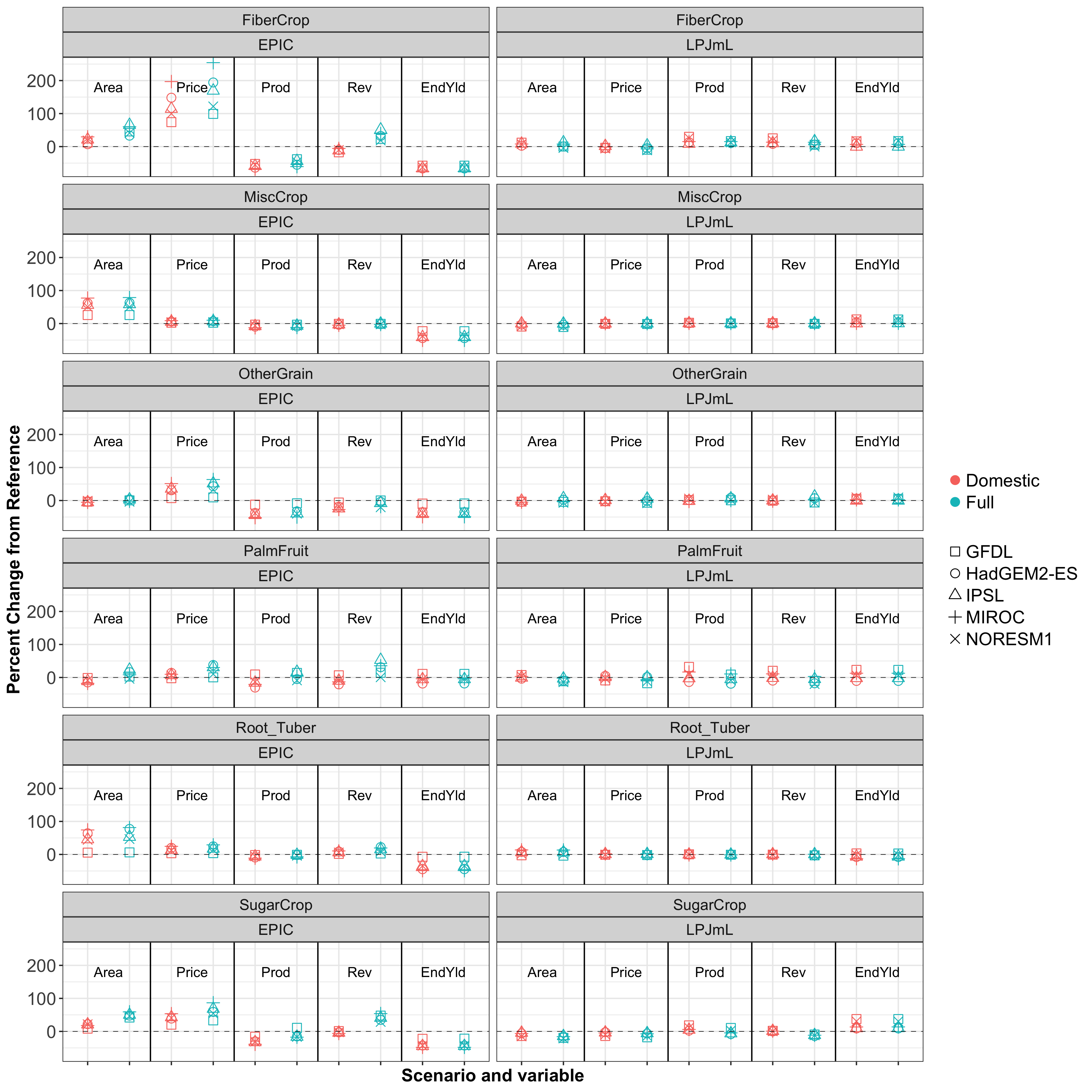

Supplement: S5 Fig — (TIFF) [file pone.0237918.s008.tiff]

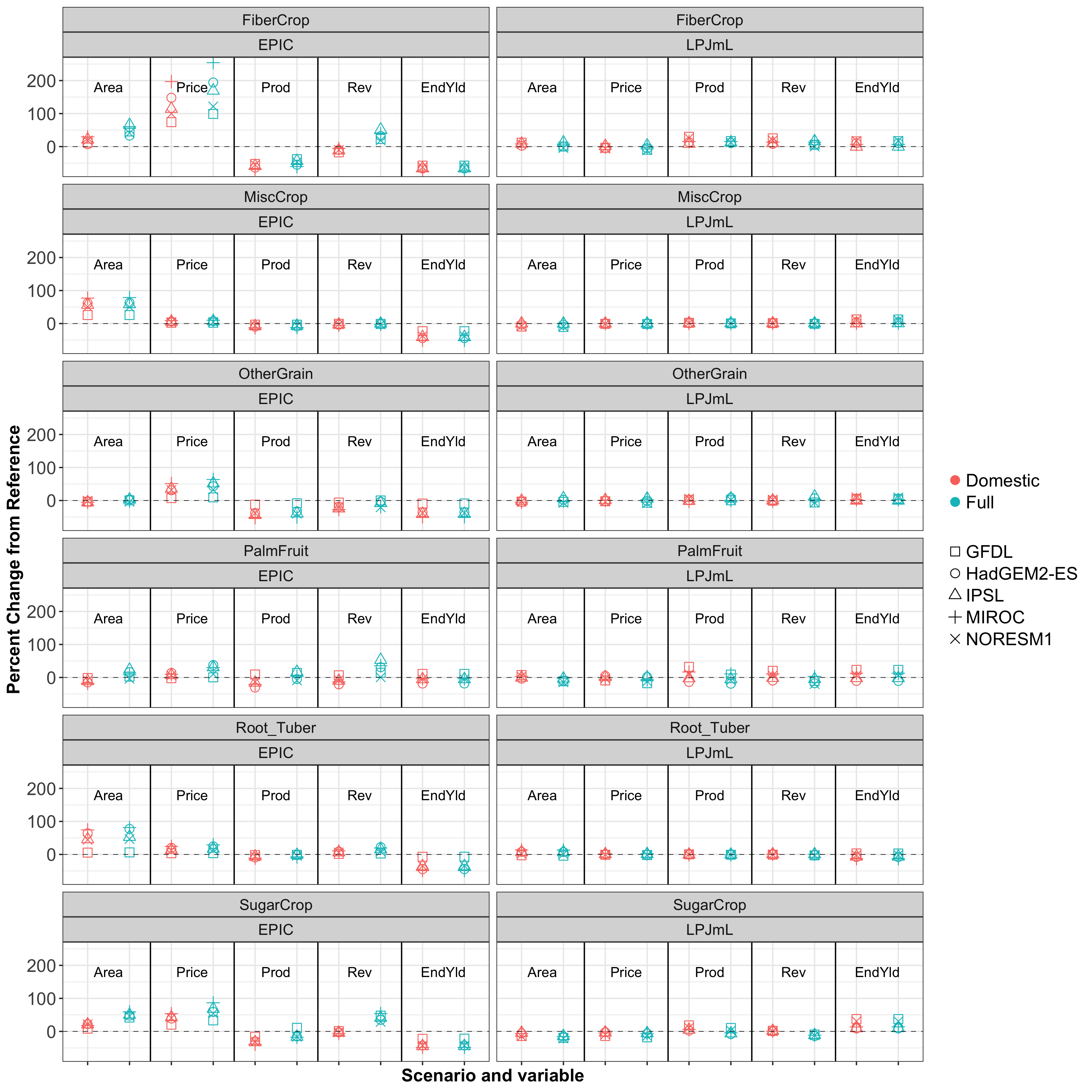

Supplement: S1 Data — (ZIP) [file pone.0237918.s009.zip › plosone-figures/figures/Fig1_2100_nonmaincrops.png]

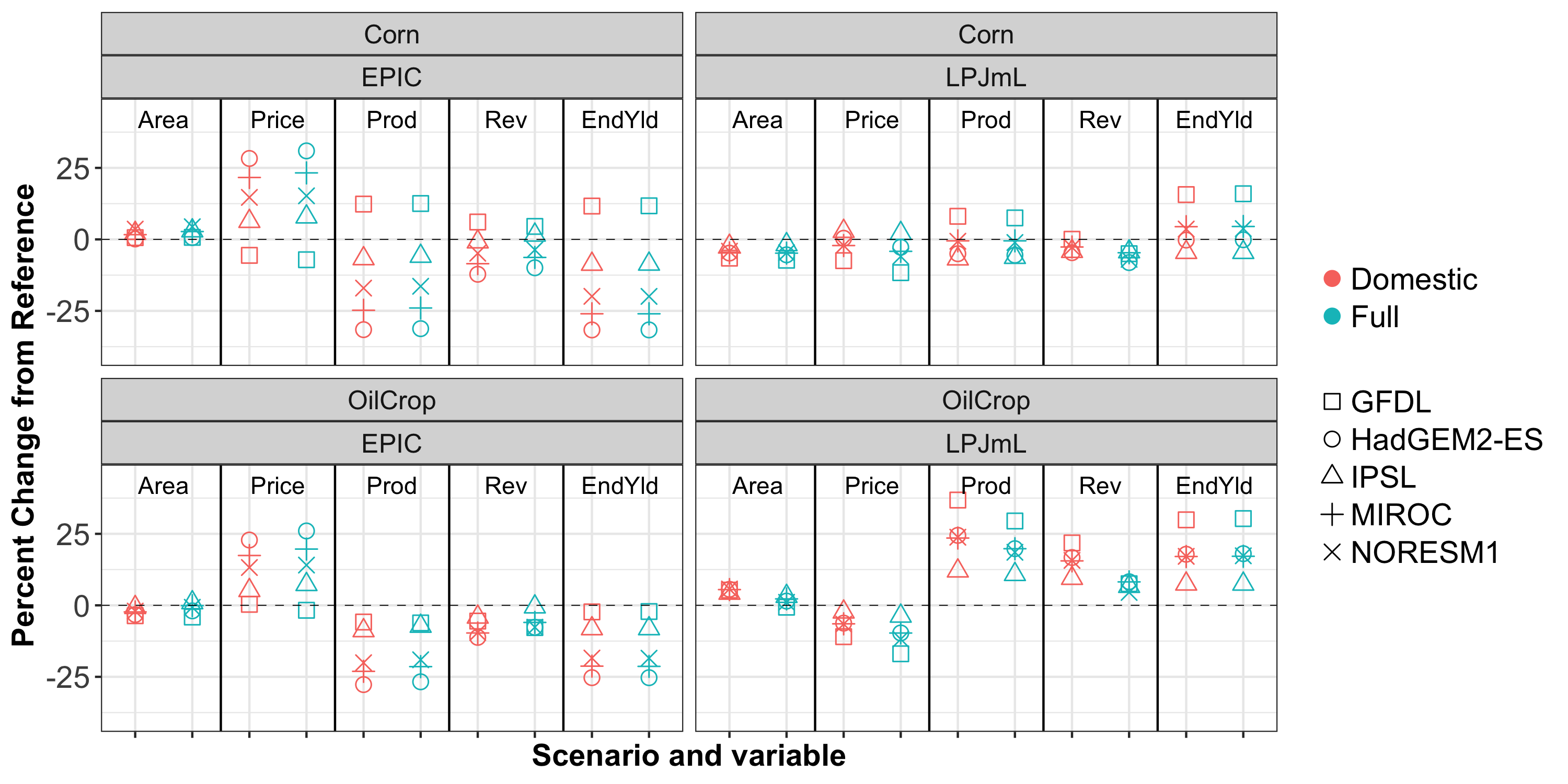

Supplement: S1 Data — (ZIP) [file pone.0237918.s009.zip › plosone-figures/figures/Fig1_2050_corn_oilcrop.png]

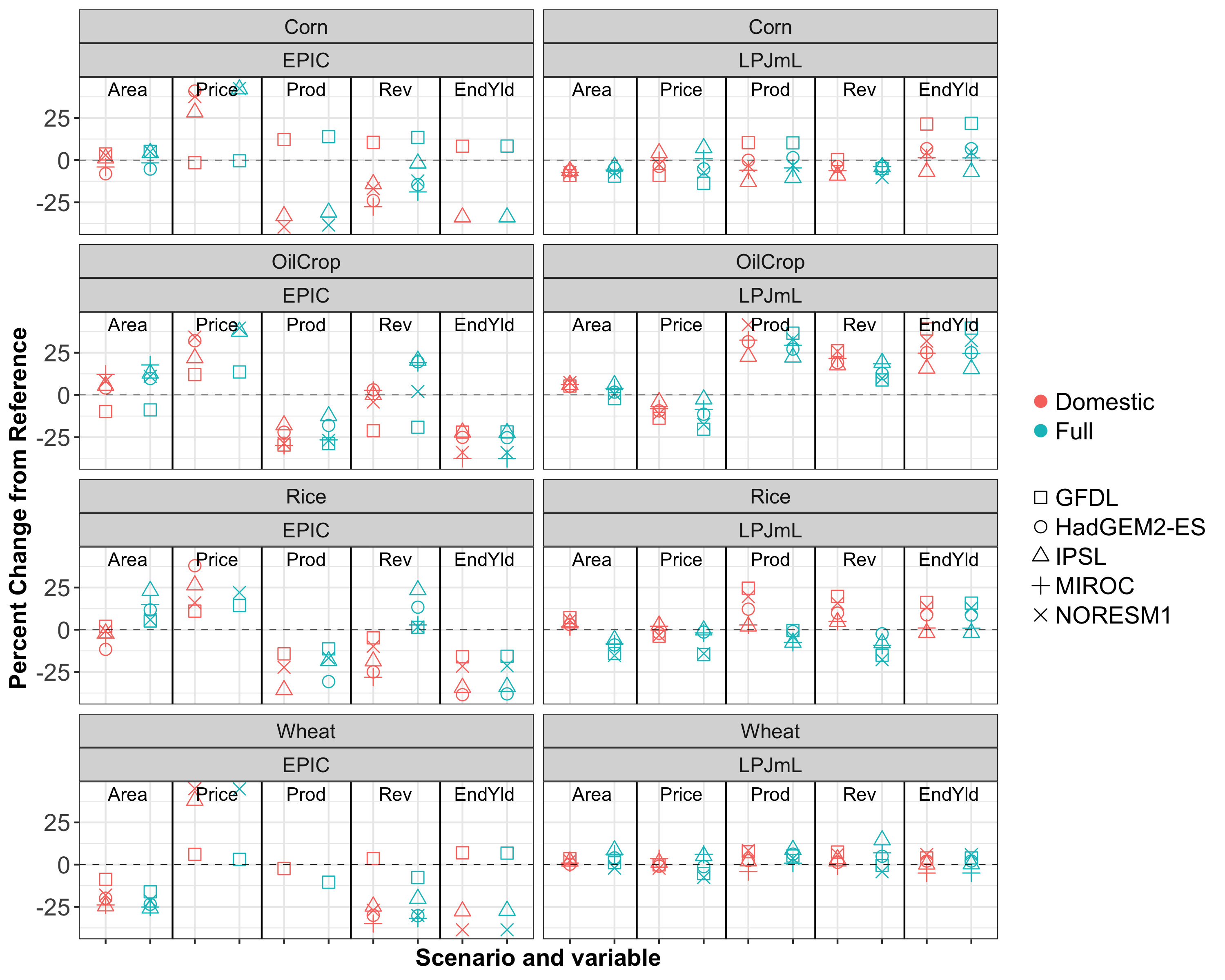

Supplement: S1 Data — (ZIP) [file pone.0237918.s009.zip › plosone-figures/figures/Fig1_2100_maincrops.png]

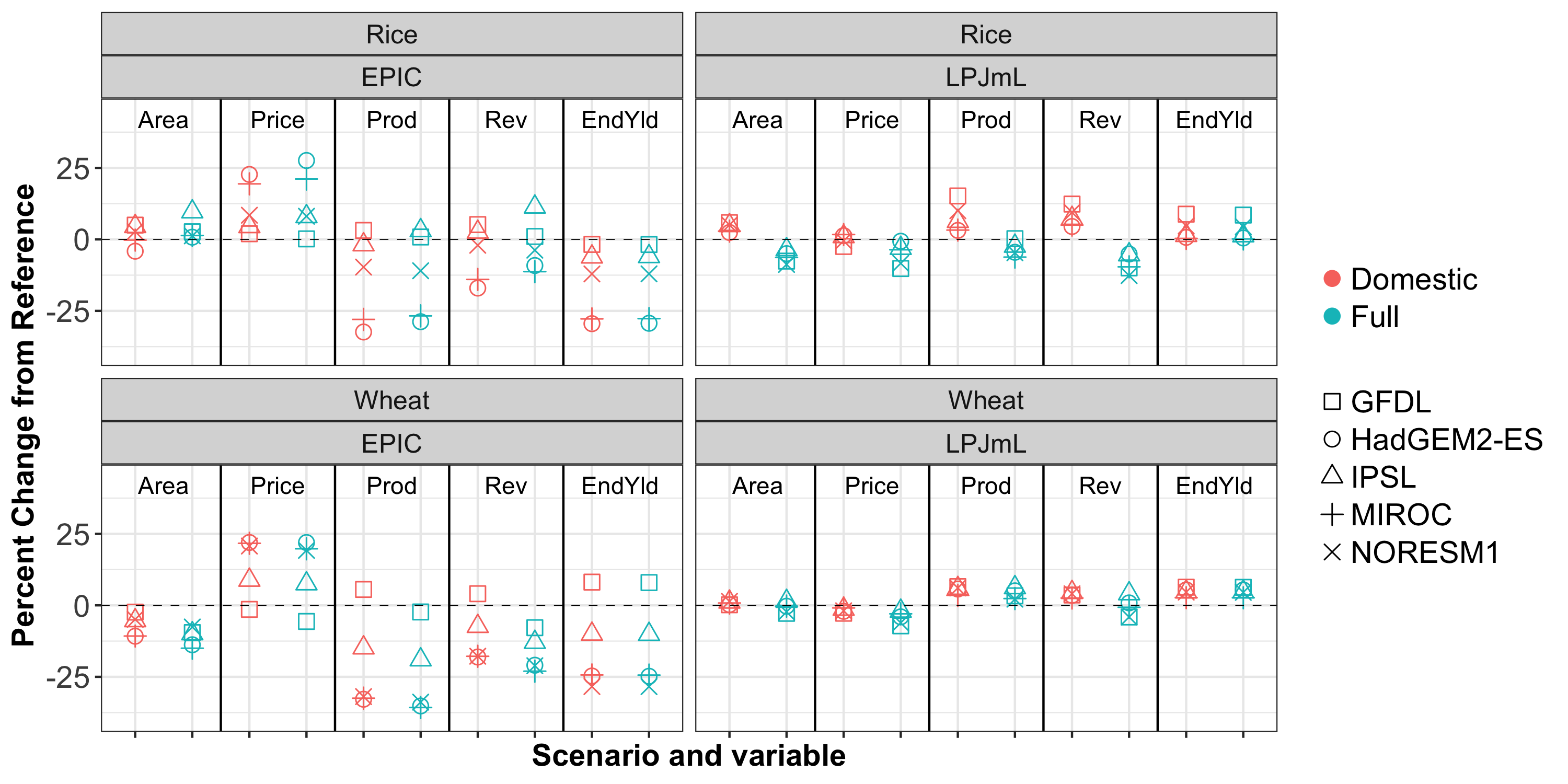

Supplement: S1 Data — (ZIP) [file pone.0237918.s009.zip › plosone-figures/figures/Fig1_2050_wheat_rice.png]

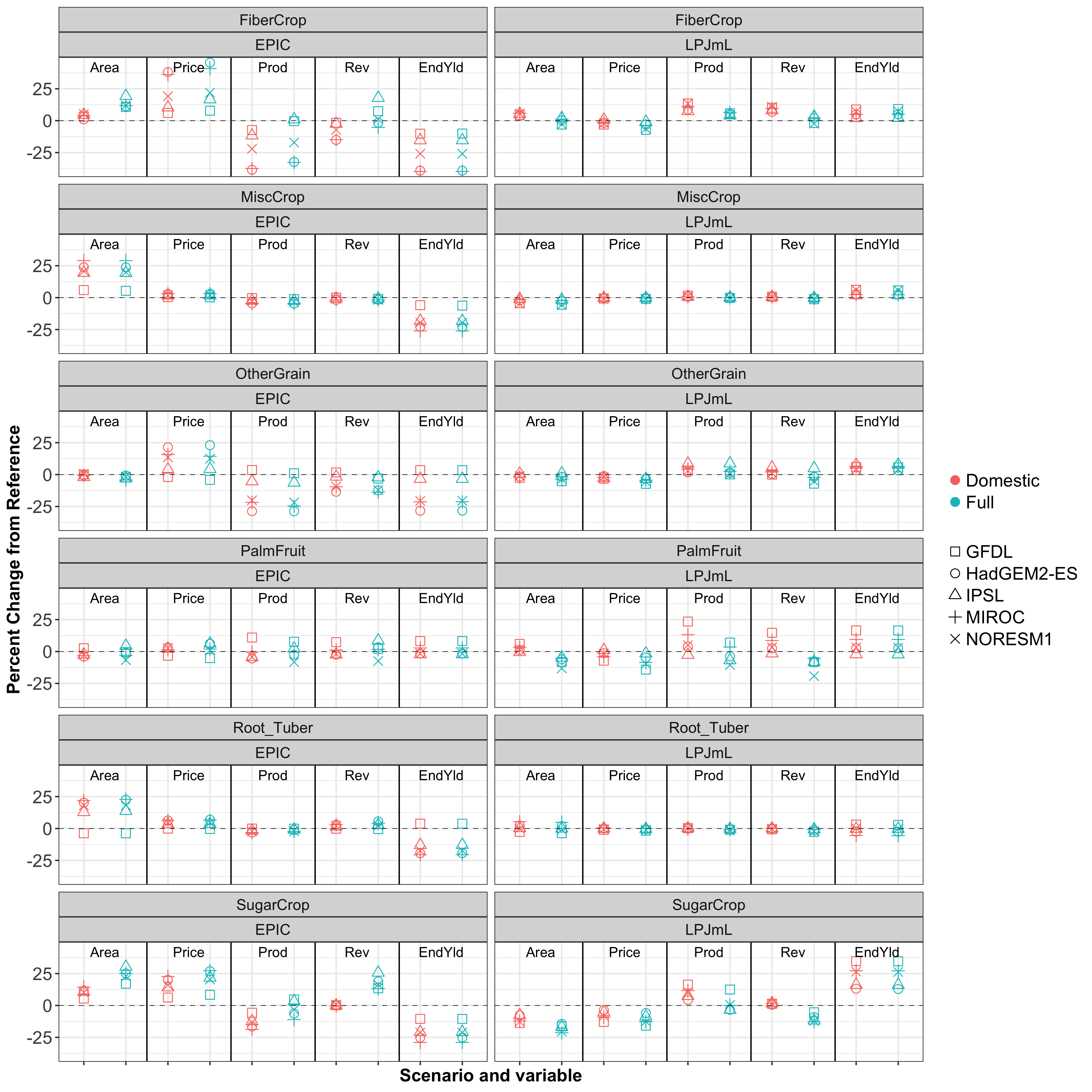

Supplement: S1 Data — (ZIP) [file pone.0237918.s009.zip › plosone-figures/figures/Fig1_2050_nonmaincrops.png]
